# Low circulating pentraxin 3 levels in pregnancy is associated with gestational diabetes and increased apoB/apoA ratio: a 5-year follow-up study

**DOI:** 10.1186/s12933-016-0345-1

**Published:** 2016-02-03

**Authors:** Tove Lekva, Annika Elisabeth Michelsen, Jens Bollerslev, Errol R. Norwitz, Pål Aukrust, Tore Henriksen, Thor Ueland

**Affiliations:** Research Institute of Internal Medicine, Oslo University Hospital, Rikshospitalet, Oslo, Norway; Mother Infant Research Institute, Tufts Medical Center, Boston, MA USA; Section of Specialized Endocrinology, Department of Endocrinology, Oslo University Hospital, Rikshospitalet, Oslo, Norway; Faculty of Medicine, University of Oslo, Oslo, Norway; Department of Obstetrics & Gynecology, Tufts Medical Center and Tufts University School of Medicine, Boston, MA USA; Section of Clinical Immunology and Infectious Diseases, Oslo University Hospital, Rikshospitalet, Oslo, Norway; Department of Obstetrics, Oslo University Hospital, Rikshospitalet, Oslo, Norway

**Keywords:** Gestational diabetes mellitus, PTX3, Cardiovascular disease, apoB/apoA ratio

## Abstract

**Background:**

Gestational diabetes mellitus (GDM) is a significant risk factor for cardiovascular disease (CVD) in later life. Pentraxin 3 (PTX3) is an essential component of innate immunity and independently associated with the risk of developing vascular events. The aim of the study was to examine the relationships between GDM, cardiovascular risk, and plasma PTX3 in pregnancy and at 5 years after the index pregnancy.

**Methods:**

This population-based prospective cohort included 300 women who had an oral glucose tolerance test (OGTT) during pregnancy. Five years later, the OGTT was repeated along with dual-energy x-ray absorptiometry, lipid analysis, and pulse wave velocity analysis. Fasting PTX3 levels were measured four times during pregnancy and at follow-up.

**Results:**

PTX3 levels were lower early in pregnancy and at 5 years follow-up in women who developed GDM. PTX3 levels throughout pregnancy were associated with body mass index. Low PTX3 levels in early pregnancy were predictive of an increased apoB/apoA ratio at 5-year follow-up. PTX3 at 5-year follow-up was inversely correlated with multiple metabolic risk factors for CVD, including body composition, arterial stiffness, dyslipidemia and previous GDM.

**Conclusions:**

Our results show that low plasma concentration of PTX3 in early pregnancy is associated with subsequent development of GDM and with an enhanced risk for CVD as estimated by an elevated apoB/apoA ratio at 5 years postpartum.

**Electronic supplementary material:**

The online version of this article (doi:10.1186/s12933-016-0345-1) contains supplementary material, which is available to authorized users.

## Background

Gestational diabetes mellitus (GDM) refers to carbohydrate intolerance first diagnosed during pregnancy. It is a condition of the pancreatic β-cells, which produce inadequate amounts of insulin to meet the increased insulin needs of late pregnancy [1]. The pathogenesis of GDM is not fully clarified, but seem to involve activation of inflammatory pathways in addition and related to metabolic and endocrine mechanisms [2]. The maternal innate immune system is stimulated during normal pregnancy, whereas the adaptive immune system is relatively suppressed. Evidence of inflammatory dysregulation (i.e., an imbalance between pro-and anti-inflammatory mediators) can be observed as early as the first trimester among pregnant women who later develop GDM [2, 3]. Subclinical inflammation is also a major risk factor for future cardiovascular disease (CVD) in the general population, and women with a history of GDM are at increased risk of CVD later in life, potentially at least partly involving inflammatory mechanisms [4]. Although several studies have demonstrated increased levels of markers reflecting vascular inflammation, such as sICAM-1 and sE-selection in women with a history of GDM [4, 5], the precise mechanism of vascular injury and CVD risk are not well understood.

Pentraxin 3 (PTX3) is an essential component of innate immunity and a member of the long pentraxin superfamily, which are soluble, multifunctional pattern recognition proteins induced by various inflammatory stimuli [6]. In contrast to the short pentraxin C-reactive protein (CRP) which is primarily produced in the liver, PTX3 is produced locally in relevant cells such as endothelial cells, macrophages and granulocytes, at the site of inflammation. Although known to be a marker of inflammation, PTX3 has also been shown to possess anti-microbial and anti-inflammatory properties, and to be cardioprotective [7, 8]. A number of anti-inflammatory molecules have been shown to modulate PTX3 expression, including high density lipoprotein cholesterol (HDL-C) in endothelial cells [6]. Knockdown of PTX3 in apolipoprotein E-knockout mice is associated with cardiac damage, inflammation and atherosclerosis pointing to a protective effect of PTX3 [9]. Thus, the enhanced levels in patients with established CVD may reflect a beneficial response, correlating with the severity of the disease, to limit the extent of immune activation [6, 10], PTX3 serum levels are inversely correlated with several features of the metabolic syndrome and obesity [11, 12].

Based on its relation to vascular inflammation, PTX3 has been suggested to be involved in vascular complication during pregnancy. Indeed, PTX3 levels are markedly elevated in women with preeclampsia [13] and type 1 diabetes during pregnancy [14], and have been shown to correlate with the degree of glucose intolerance in women with GDM [15, 16]. However, PTX3 levels have not been prospectively evaluated during pregnancy and related to future CVD risk. As GDM women are more prone to develop CVD later in life and our overarching hypothesis is that this enhanced risk may start to develop or accelerate during pregnancy. We therefore measured circulating PTX3 in 300 women from a prospective cohort study at multiple times during pregnancy and at 5-year follow-up. We hypothesized that GDM women would be present with regulated PTX3 levels and would be associated with CVD risk as reflected by lipid ratios at 5 years follow-up.

## Methods

### Study population

The STORK study was a prospective cohort study with a longitudinal design in which 1031 low-risk women of Scandinavian heritage who gave birth at Oslo University Hospital Rikshospitalet between 2002 and 2008 were followed throughout their pregnancy. Exclusion criteria included multiple pregnancy, known pre-gestational diabetes, severe chronic medical conditions (such as lung, cardiac, gastrointestinal or renal diseases), and pregnancies complicated by major fetal malformations. Details about the study have been previously published [17]. Briefly, each pregnant woman had four antenatal visits at gestational age (GA) weeks 14–16, 22–24, 30–32, and 36–38. Clinical data and blood samples were collected at each visit, processed, and stored at −80 °C until further analysis. A 75 g oral glucose tolerance test (OGTT) was performed on all women at antenatal GA visit 30–32 weeks.

The current study is a 5-year follow-up after the index pregnancy [18]. A total of 1031 participants from the original STORK cohort were invited to participate; the 10 women who developed preeclampsia in the index pregnancy (including two who developed both GDM and preeclampsia) were not included in this analysis to avoid the obvious confounder of preeclampsia. Exclusion criteria included pregnancy at the time of invitation and/or delivery within the past year. Three hundred women agreed to participate. Written informed consent was obtained from all participants. All clinical investigations were conducted according to the principles in the Declaration of Helsinki. The study was approved by the Regional Committee for Medical Research Ethics of Southern Norway in Oslo, Norway.

At the time of the 5-year follow-up visit, a fasting blood draw was performed to measure lipid profiles and a 75 g OGTT was conducted. For the purposes of this analysis, the term primiparous is used to identify women delivering their first child in the index pregnancy (nulliparous) or with only one prior delivery at 5-year follow-up.

### Measurements of glycemic and lipid parameters

All 75 g OGTTs were performed in the morning after an overnight fast. Venous EDTA blood was analyzed at point of care using an Accu-Check Sensor glucometer (Roche Diagnostics GmbH, Mannheim, Germany). Additional venous blood samples were allowed to clot for 30 min and the serum separated by centrifugation for 10 min at 3000*g* and stored at −80 °C. Glucose levels were also measured from frozen serum samples collected at 30–32 weeks using the hexokinase method (Hitachi Modular P800, Roche Diagnostics, Mannheim, Germany) at an accredited clinical chemistry laboratory at Oslo University Hospital Rikshospitalet, as previously reported [18]. For the 5-year follow-up study, we used the glucose data from the Accu-check Sensor glucometer (Roche Diagnostics, Mannheim, Germany). Insulin levels in the stored samples were assayed in duplicate by RIA (Diagnostic Products Corporation, Los Angeles, CA, USA), as previously reported [18]. Levels of apolipoprotein A (apoA), apoB, HDL-C, low density lipoprotein cholesterol (LDL-C) (directly measurements), and triglycerides (TG) were measured from frozen serum samples at follow-up at an accredited clinical chemistry laboratory at Oslo University Hospital Rikshospitalet. The ratios of TG/HDL-C and apoB/apoA are known risk factors for CVD [19, 20], and were calculated based on the above measurement. For PTX3 and CRP analysis, we used fasting plasma from venous EDTA blood sampled on ice, centrifuged for 25 min at 3000*g* at 4 °C, separated, and stored at −80 °C until analyzed. PTX3 and CRP levels were measured in duplicate using a commercially available enzyme-linked immunosorbent assay (ELISA; R and D Systems, Minneapolis, MN, USA) in a 384 format using the combination of a SELMA (Jena, Germany) pipetting robot and a BioTek (Winooski, VT, USA) dispenser/washer. Absorption was read at 450 nm with wavelength correction set to 540 nm using an ELISA plate reader (Bio-Rad, Hercules, CA, USA).

### Diagnosis of GDM

GDM was diagnosed on a 75 g OGTT using both the new IADPSG criteria and the old WHO criteria as follows: (1) IADPSG criteria: fasting plasma glucose (FPG) of 5.1–6.9 mmol/L and 1 h plasma glucose ≥10.0 mmol/L or 2 h plasma glucose 8.5–11.0 mmol/L; and (2) WHO criteria: 2 h plasma glucose ≥7.8 mmol/L [21]. Insulin sensitivity was measured on the same samples collected at the time of OGTT using the Matsuda index (i.e., 10,000/square root of [fasting glucose (mmol/L) × fasting insulin (mU/L)] × [mean glucose (mmol/L) × mean insulin (mU/L)]) during OGTT. This index is a measure of whole body insulin sensitivity that has been validated against the euglycemic-hyperinsulinemic clamp [22]. β-cell function was assessed with the insulin secretion-sensitivity index (ISSI-2) [area under the curve (AUC) insulin (mU/L)_0-120_/glucose (mmol/L)_0-120_ × Matsuda], which has been validated against the disposition index from the intravenous GTT [23]. Homeostasis model assessment: insulin resistance(HOMA–IR) was calculated as fasting insulin (mU/L) × fasting glucose (mmol/L)/22.5, as previously described by Matthews et al. [24]. The women diagnosed with GDM were not using any anti-diabetic medicine.

### Measurements of arterial stiffness

All participants were examined at the 5-year follow-up visit on the morning after fasting overnight. Aortic stiffness was assessed by means of PWV measurements using SphygmoCor (Atcor Medical, Sydney, Australia), a non-invasive technique with direct-contact pulse sensors. Aortic PWV was measured by sequential recordings of the arterial pressure waveform at the carotid and femoral arteries. The PWV was calculated as the distance between recording sites measured over the surface of the body (*L*), divided by the time interval (*t*) between the feet of the flow waves (PWV  =  *L*/*t*). The value was averaged over 10 cardiac cycles [25]. Only measurements that met the automatic quality control cutoff were used in the final analysis. Average SD of all measurements (mean time difference between carotid and femoral) was below 5 %. All measurements were performed by the author (TL), and we have no interobserver variability.

### Measurements of body fat composition

Total body composition was determined by dual-energy x-ray absorptiometry (DXA; GE Lunar Prodigy Densitometer (software version 12.10), GE Medical Systems, Lunar Corp., Madison, WI, USA) and analyzed using enCORE software (version 14.10; GE Medical Systems), as previously described [18]. All DXA scans were performed by the author (TL). CoreScan has been previously validated against volumetric computed tomography [26, 27]. For measuring android fat, a region of interest (ROI) was defined with the caudal limit at the top of the iliac crest and the cephalic limit at the base of the skull. Android ROI contains both visceral (VAT) and subcutaneous adipose tissue (SAT). The software estimates the quantity of SAT in the android ROI. VAT was computed by subtracting SAT from the total android fat. The fat mass data from DXA was transformed to volume using a constant correction factor (0.94 g/cm^3^) consistent with the density of adipose tissue [26]. All VAT under 50 g was set to 50 g since the DXA measurement is unreliable in the low range visceral fat content [28].

### Statistical analysis

Statistical analyses were conducted using SPSS for Windows, version 21.0 (Chicago, IL, USA). Data are expressed as mean ± SD when normally distributed and median (25th, 75th percentile) when skewed. Comparison between women with and without a history of GDM was performed using *t* test or Mann–Whitney U depending on distribution, and Chi square test for categorical variables. Univariate and stepwise (probability of F to-enter 0.1 -remove 0.15) linear regression analyses were carried out on log transformed variables (if skewed) and results given as standardized regression coefficients. Only variables below p < 0.2 were included in the stepwise multivariable models. Logistic regression was used to calculate odds ratios for risk factors according to established cut-offs for the apoB/apoA and TG/HDL-C ratio. Two-tailed p values <0.05 were considered significant.

## Results

Table 1 shows the characteristics of the study population during the index pregnancy and at the time of the 5-year follow-up visit stratified into those women who did and did not have GDM in the index pregnancy using both the IADPSG (50 with and 234 without GDM) and WHO criteria (31 with and 253 without GDM), based on the OGTTs. As evident from Table 1, women with GDM based on the IADPSG criteria were on average older and had a higher body mass index (BMI) both in the index pregnancy and at follow-up, while women with GDM based on the WHO criteria had a higher BMI at follow-up compared to their non-GDM counterparts. Women belonging to both criteria were more frequently smokers at follow-up.

**Table 1:** Characteristics of the study population according to the new GDM IADPSG criteria and the old GDM WHO criteria

Variable		Visit 3 (week 30–32) in the index pregnancy		Follow-up visit	
		GDM	Non-GDM	GDM	Non-GDM
N=	IADPSG	50	234	50	234
	WHO	31	253	31	253
Follow-up time (years)	IADPSG			5.1 (4.6, 5.3)	4.8 (4.4, 5.4)
	WHO			5.0 (4.5, 5.4)	4.8 (4.4, 5.4)
Age (years)^a^	IADPSG	33.6 ± 4.3	32.0 ± 3.7*	38.9 ± 4.4	37.4 ± 3.7*
	WHO	33.1 ± 3.7	32.2 ± 3.8	38.6 ± 3.8	37.5 ± 3.8
Height (cm)^a^	IADPSG	169 ± 6	169 ± 6	168 ± 6	169 ± 6
	WHO	168 ± 5	169 ± 6	168 ± 5	169 ± 6
BMI (kg/m^2^)	IADPSG	28.2 (26.8, 30.8)	26.2 (23.7, 28.4)**	24.7 (22.5, 28.0)	22.6 (20.8, 24.6)**
	WHO	27.8 (25.7, 31.2)	26.4 (23.9, 28.6)	24.1 (21.7, 28.1)	22.8 (20.9, 25.1)*
Primipara n (%)	IADPSG	22 (44.0)	118 (51.1)	6 (12.0)	26 (11.1)
	WHO	18 (60.0)	122 (48.6)	6 (19.3)	26 (10.3)
Family history heart disease n (%)	IADPSG			31 (64.5)	134 (57.7)
	WHO			22 (75.9)	143 (57.0)
Family history diabetes n (%)	IADPSG			17 (34.0)	71 (30.3)
	WHO			13 (41.9)	75 (29.6)
Currently smoking n (%)	IADPSG	1 (2.0)	7 (3.0)	13 (26.0)	35 (15.0)*
	WHO	1 (3.2)	7 (2.8)	9 (29.0)	39 (15.4)*
Previous smoker n (%)	IADPSG	14 (28.0)	39 (16.7)	15 (30.0)	50 (21.3)
	WHO	8 (25.8)	45 (17.8)	8 (25.8)	57 (22.5)
Systolic blood pressure (mmHg)	IADPSG	115 (105, 120)	110 (105, 120)	110 (100, 130)	110 (100, 120)
	WHO	110 (100, 120)	110 (105, 120)	110 (100, 130)	110 (100, 120)
Diastolic blood pressure (mmHg)	IADPSG	70 (60,73)	70 (60, 70)	70 (65, 75)	70 (60, 75)
	WHO	70 (60, 70)	70 (60, 70)	70 (65, 80)	70 (60, 75)
Mean arterial pressure (mmHg)	IADPSG	83.3 (78.3, 88.3)	81.7 (76.7, 86.7)	83.3 (76.7, 92.1)	83.3 (76.7,88,3)
	WHO	82.5 (77.5, 86.7)	83.3 (76.7, 86.7)	83.3 (78.7, 95.0)	83.3 (76.7, 88.3)
Pulse pressure (mmHg)	IADPSG	45 (40,50)	43 (40,50)	40 (40,50)	40 (40,50)
	WHO	40 (40,50)	45 (40,50)	40 (35,50)	40 (40,50)

^a^Visit 1. Data given as mean ± SD when normal distributed and median (25th, 75th) when skewed distributed. Comparison between women with GDM and non-GDM were performed using t test for normal distributed variables, Mann–Whitney U for non-distributed continuous variables, and Chi test for categorical variables

* p < 0.05

** p < 0.001

### Women with GDM have lower plasma PTX3 in early pregnancy and at follow-up compared to non-GDM women

**Fig. 1:**
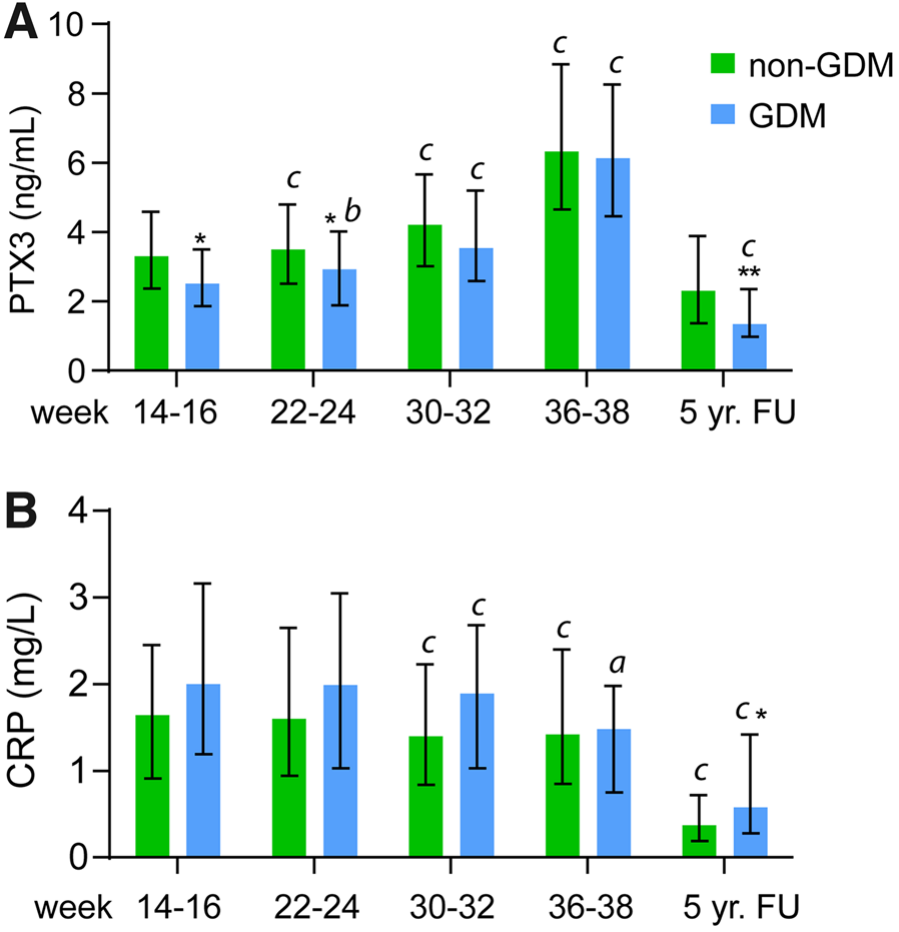
Circulating **A** PTX3 and **B** CRP levels in pregnancy and at 5-year follow-up between GDM (WHO) and non-GDM. FU, follow-up. Date is given as median and 25th/75th percentile. *p < 0.05, **p < 0.01 compared with non-GDM group. ^a^p < 0.05, ^b^p < 0.01, ^c^p < 0.001 vs. week 14–16 within group

Plasma PTX3 levels increased during pregnancy in both GDM and non-GDM women from 14–16 weeks to 36–38 weeks (p < 0.001 for all time points), and was lower at 5 years follow-up compared to during pregnancy (Fig. 1A). While this pattern was seen in the study group as a whole, women with GDM had significantly lower PTX3 levels than those without GDM at 14–16 weeks and 22–24 weeks as well as at 5-year follow-up when using the WHO criteria, but only at 5-year follow-up when using the IADPSG criteria (Additional file 1: Table S1). For CRP, no differences between GDM and non-GDM women using WHO criteria was observed during pregnancy, but GDM women had higher CRP at 5 years follow-up (Fig. 1B). However, using the IADPSG criteria, GDM women had higher CRP levels at 14–16, 22–24 and 30–32 weeks (Additional file 1: Table S1). We have previously demonstrated enhanced CVD risk in GDM women based on the WHO criteria compared to the IADPSG criteria [29]. Subsequent analysis was therefore restricted to women diagnosed with GDM based on the WHO criteria. Consistent with prior reports [13], we also found that women with preeclampsia in the index pregnancy (n = 10) had markedly higher circulating PTX3 levels, but not CRP levels at all time points in pregnancy; PTX3 (median 25th, 75th); 3.85 (3.49, 6.42) ng/mL at 14–16 weeks, 5.03 (3.71, 6.19) ng/mL at 22–24 weeks, 7.33 (5.45, 10.40) ng/mL at 30–32 weeks, and 11.49 (6.73, 17.71) ng/mL at 36–38 weeks (p < 0.05 compared to non GDM), but not at 5-year follow up [3.53 (2.28, 4.82) ng/mL; p = 0.16]. Women with preeclampsia were excluded from further analysis.

### Associations between PTX3 and clinical characteristics in pregnancy

We next evaluated the association between PTX3 levels and clinical variables at each of the four antenatal visits in pregnancy (Table 2), and found that BMI was consistently negatively associated with PTX3 levels at each visit. Systolic blood pressure (BP) was inversely associated with PTX3 levels at 30–32 weeks. No associations were found between PTX3 levels and CRP, parity or smoking status prior to pregnancy. Indices of glucose metabolism calculated from the OGTT at 30–32 weeks revealed that PTX3 levels was positively associated with insulin sensitivity (r = 0.23, p < 0.001) and β-cell function (r = 0.18, p = 0.003), and negatively with insulin resistance (r = −0.23, p < 0.001). Conversely, CRP was consistently positively associated with BMI at all time-points during pregnancy (r = 0.25–0.27, p < 0.001) and insulin resistance at 30–32 weeks (r = 0.25, p < 0.001) and negatively associated with insulin sensitivity (r = −0.28, p < 0.001) and β-cell function (r = −0.14, p = 0.021).

**Table 2:** Association between PTX3 levels in pregnancy and clinical characteristics throughout pregnancy

Variables	14–16 weeks		22–24 weeks		30–32 weeks		36–38 weeks	
	r	p	r	p	r	p	r	p
Age	−0.01	0.851	−0.01	0.982	0.05	0.422	0.03	0.625
BMI	−0.21	<0.001	−0.23	<0.001	−0.18	0.002	−0.23	<0.001
CRP	0.07	0.261	0.05	0.402	−0.03	0.664	0.00	0.968
Parity^a^	−0.03	0.581	−0.03	0.636	0.01	0.911	−0.03	0.587
Smoking^b^	−0.08	0.206	−0.02	0.743	−0.03	0.651	−0.03	0.606
Systolic BP	−0.06	0.289	−0.06	0.328	−0.16	0.006	−0.03	0.595
Diastolic BP	−0.02	0.689	−0.06	0.261	−0.05	0.418	0.05	0.428

^a^ Primipara/multipara

^b^ Previous and current

### Associations between PTX3 levels and cardio-metabolic risk markers at 5-year follow-up

As shown in Table 3, and observed during pregnancy PTX3 levels were negatively correlated with BMI and showed a similar association with VAT. Further, PTX3 was positively correlated with insulin sensitivity and negatively correlated with insulin resistance, although these associations were modest. We recently demonstrated that these GDM women are characterized by dyslipidemia and increased arterial stiffness [29]. PTX3 levels were negatively correlated with arterial stiffness, apoB and LDL-C, and positively correlated with the atheroprotective lipids apoA and HDL-C. No association was found between PTX3 and CRP. Thus, circulating PTX-3 may reflect a composite of different factors associated with metabolic and in particular cardiovascular risk.

**Table 3:** Associations between PTX3 and cardio-metabolic risk factors 5-year follow-up

Variables	Univariate	
	r	p
Follow-up	0.04	0.516
Age	0.01	0.997
Diabetes in family	0.06	0.601
Heart disease in family	−0.07	0.507
BMI	−0.29	<0.001
Parity^a^	0.05	0.437
Smoking^b^	0.10	0.105
GDM (WHO criteria)	−0.16	0.006
Systolic BP (mmHg)	−0.12	0.055
Diastolic BP (mmHg)	−0.05	0.447
Insulin sensitivity	0.13	0.033
Insulin resistance	−0.12	0.046
β-cell function	0.11	0.063
PWV	−0.18	0.002
apoA	0.22	<0.001
apoB	−0.25	<0.001
LDL	−0.23	<0.001
TG	−0.10	0.082
HDL	0.22	<0.001
CRP	0.05	0.405
Visceral fat	−0.29	<0.001

^a^Primipara/multipara

^b^Previous and current

### Predictors of CVD risk as reflected by lipid and apolipoprotein ratios

**Fig. 2:**
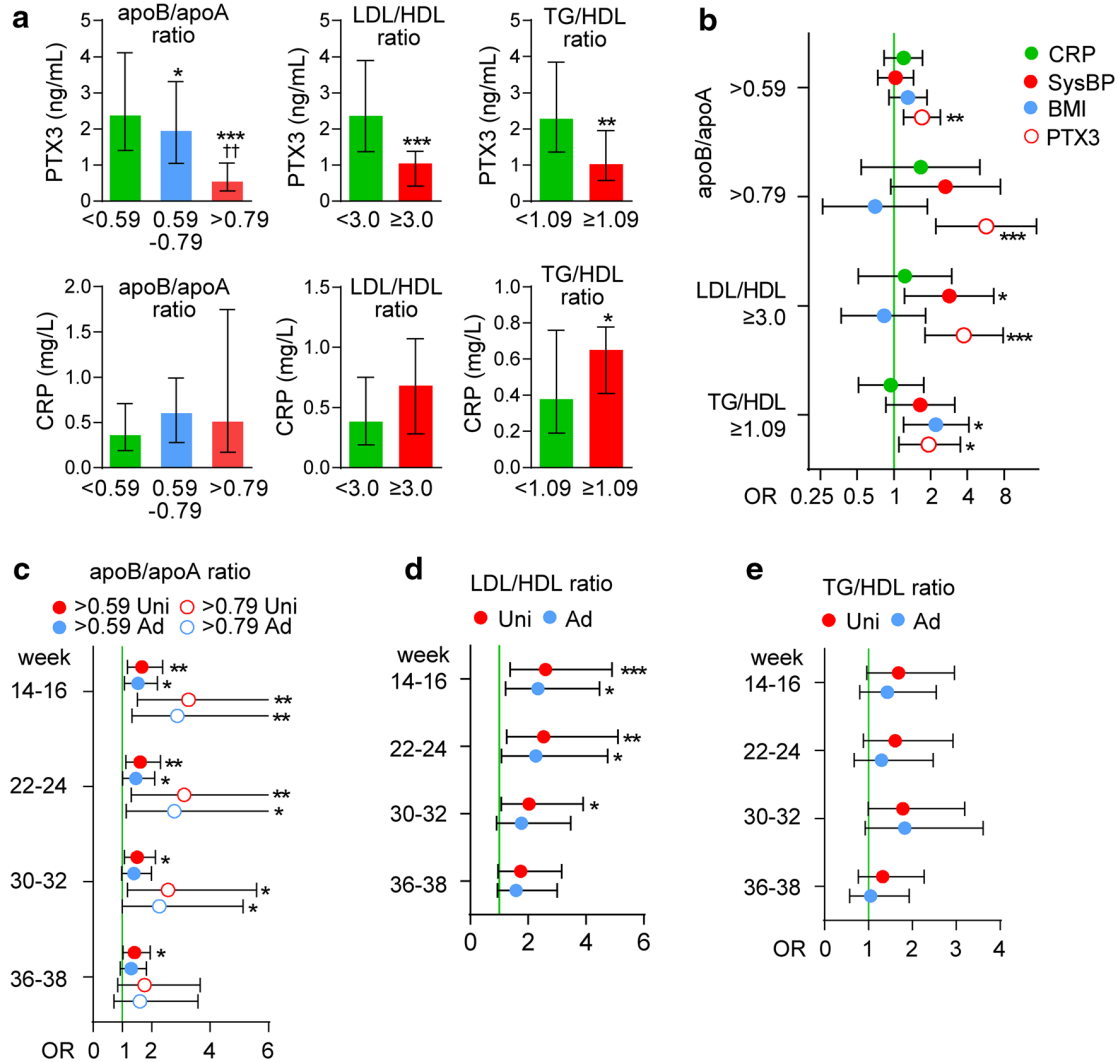
Low circulating PTX3 during pregnancy and at 5-year follow-up is associated with increased cardiovascular disease (CVD) risk. **a** PTX3 and CRP levels at follow-up according the TG/HDL-C ratio representing increased CVD risk (>1.09), LDL/HDL-C ratio >3.0 and apoB/apoA ratio representing low (<0.60), moderate (≥ 0.60–0.79) and high risk (≥0.80) for coronary artery disease (CAD). *p < 0.05, **p < 0. 01, ***p < 0.001 vs. reference group (*green*); ^††^p < 0.01 vs. intermediate risk (≥0.59 <0.79). **b** Adjusted risk models for PTX3, BMI, systolic BP and CRP at follow-up for moderate and high risk as reflected by apoB/apoA ratio, LDL/HDL-C ratio and the TG/HDL-C ratio according to cut-offs in A. **c** Univariate (*red circles*) and adjusted (*blue circles*) models for moderate and increased CVD risk as reflected by apoB/apoA ratios by PTX3 during pregnancy. **d** Univariate (*red circles*) and adjusted (*blue circles*) models for increased CVD risk as reflected by LDL/HDL-C ratio by PTX3 during pregnancy. **e** Univariate (*red circles*) and adjusted (*blue circles*) models for increased CVD risk as reflected by the TG/HDL-C ratio by PTX3 during pregnancy. The adjusted analysis included BMI and systolic BP acquired at the same time as the PTX3 measurement. PTX3, BMI, systolic BP and CRP are expressed as log change per SD. For PTX3 the inverse function is given to reflect increasing risk with lower levels. *p < 0.05, **p < 0. 01, ***p < 0.001 vs. reference group

As shown above, apoA and apoB were asssociated with PTX3 at 5-year follow-up (Table 3). The apoB/apoA ratio is a well-established biomarker for the development of atherosclerotic disorders as well as adverse CVD outcome [19]. In addition, we have recently demonstrated that GDM women have significantly higher TG/HDL-C ratios, a biomarker that may reflect enhanced risk of metabolic complications and risk of CVD [29]. The LDL/HDL ratio is also a CVD risk indicator, with a better predictive value than isolated parameters used independently, particularly LDL [30]. We therefore next investigated if PTX3 was associated with these ratios using an apoB/apoA ratio of >0.59 to define moderate-risk and >0.79 to define high-risk [19], a cut-off of >3.0 for LDL/HDL [30] and a cut-off of >1.09 for the TG/HDL ratio [20]. As shown in Fig. 2a, a stepwise risk increase using the apoB/apoA ratio as a proxy for CVD was observed with decreasing PTX3 levels at 5-year follow-up. Similar but more modest difference was observed for the LDL/HDL and TG/HDL-C ratios (Fig. 2a). Additional file 1: Table S2 shows the univariate odds ratios (ORs) for PTX3, CRP and other CV risk factors at 5 years follow-up in relation to these ratios. In addition to PTX3, the strongest predictors for CVD risk were BMI and systolic BP, with some associations for parity and smoking on some ratios. Figure 2b shows multivariable models and demonstrates that, at 5-year follow-up, PTX3 remains significantly associated with CVD risk even after accounting for BMI and systolic BP. No association between CRP and these ratios was observed.

### Prediction of CVD risk at 5-year follow-up by PTX3 levels during the index pregnancy

We finally assessed if circulating PTX3 at different time-points during pregnancy could predict moderate or high CVD risk, based on apoB/apoA ratio, in univariate and multivariable analysis, adjusting for BMI and systolic BP, acquired at the time of PTX3 measurement. As shown in Fig. 2c, low PTX3 levels were associated with moderate-risk and especially high-risk for CVD from 14–16 weeks until 30–32 weeks, with only a minor effect of adjustment. A similar patterns was observed for the LDL/HDL ratio (Fig. 2d). When evaluating CVD risk as reflected by the TG/HDL ratio no association with PTX3 levels during pregnancy was found (Fig. 2e). The ORs for these associations are given in Additional file 1: Table S3. ROC curves with AUC data for PTX3 and prediction of apoB/apoA and the LDL/HDL ratio at 5 years follow-up are given in Additional file 1: Figure S1. For the early time-points, PTX3 gives good discrimination (AUC 0.77–0.81) of high CV risk according to the apoB/apoA ≥0.79, which supports the conclusion from the univariate and multivariable analysis. The discrimination for the LDL/HDL ratio ≥3.0 gave a more modest discrimination.

## Discussion

Our study demonstrates that circulating PTX3 levels are lower early in pregnancy and at 5-year follow-up in women who have GMD as compared with those who do not. PTX3 was consistently negatively correlated with BMI during pregnancy, and was associated with multiple metabolic risk factors for CVD at 5-year follow-up. Low PTX3 levels in pregnancy were correlated with enhanced future CVD risk as estimated by the apoB/apoA and LDL/HDL ratio at 5-year follow-up, and this association was independent of BMI and CRP. Our findings suggest that low PTX3 levels early in pregnancy, and in particular in GDM could confer enhanced long-term CVD risk.

### Comparison with previous studies

A major finding in our study was that PTX3 levels were decreased in women with GDM compared to non-GDM women, in particular at 5-year follow-up. This is in contrast to two recent studies showing no difference or increased PTX3 levels in GDM and compared with non-GDM women. The reason for this discrepancy is unclear, but could involve a lower number of patients with GDM and in particular of controls in the two other studies, and notably in one of the studies there was no differences in lipid values, BMI or measures of glucose metabolism between GDM and non-GDM women suggesting that they are studying a milder GDM phenotype, which is also supported by the GDM diagnostic criteria used (ADA and NDDG) [15, 16]. Furthermore, while Yilidrim et al. [16] observed a positive correlation between PTX3 levels and response to the glucose challenge test, we found positive correlations with β cell function and insulin sensitivity and a negative correlation with insulin resistance, suggesting a potential beneficial effect of PTX3. A protective role for PTX3 in normal pregnancy, with increased levels closer to gestation, as observed in our study, has been reported in several other publications [31, 32].

Although not the focus of our study, we also observed markedly enhanced PTX3 levels in preeclamptic women at all time points, supporting a number of publications showing that PTX3 is closely correlated with the severity and progression of preeclampsia [13]. This may reflect a feedback mechanism aimed at counterbalancing overactive pro-inflammatory signals [6, 8], and some studies have suggested that the increased levels of PTX3 observed in preeclampsia may have a placental origin coming from the increased number of macrophages in the placental bed [13].

### PTX3 and metabolic profile

Obesity and GDM are both chronic low-grade inflammatory states [33] and elevated circulating markers of endothelial dysfunction are present in young females with a history of GDM [34]. This chronic inflammatory state appears to trigger insulin resistance in the skeletal muscles of obese individuals. We have previously shown that the GDM women have higher BMI and visceral fat content at 5-year follow-up [18]. The negative correlation between BMI and PTX3 levels during pregnancy and at 5-year follow-up supports recent studies demonstrating low PTX3 levels in patients with the metabolic syndrome and obesity [11, 35]. Furthermore, recent studies investigating the association between plasma PTX3 levels and insulin resistance in lean, overweight, and obese individuals [35, 36] supports our data of a negative correlation with glucose intolerance. Miyaki et al. [37] showed that PTX3 production in adipose tissue and skeletal muscles of diabetic obese mice was significantly lower than in control mice suggesting that PTX3 may play a role in promoting insulin sensitivity by regulating glucose transport proteins. It is tempting to hypothesize that similar mechanisms could be operating in women with GDM. The observation that women diagnosed with GDM based on WHO criteria are the subjects with the lowest PTX3 levels during pregnancy and follow-up, may thus indicate that low PTX3 might be a marker of more severe metabolic impairment and cardiovascular risk.

### Cardioprotective effects of PTX3

We have recently demonstrated that women with GDM have a moderately increased arterial stiffness, an early indicator of CVD risk, at 5-year follow-up [29]. Circulating PTX3 is now accepted as a major CVD risk factor, providing independent prognostic information in populations with established CVD. However, the precise role of PTX3 in vascular diseases is unknown and its relevance in patients without manifest CVD is less well established. Furthermore, several experimental models and in vitro studies support a cardioprotective function of PTX3 [7]. Thus, PTX3 deficiency is associated with increased inflammation, cardiac damage, and atherosclerosis. PTX3 deficient mice develop larger atherosclerotic lesions compared to controls [6]. In addition, PTX3 may inhibit complement activation and increase the progression of atherosclerosis [38]. Our finding of a negative correlation between PTX3 levels and arterial stiffness as estimated by PWV, and as previously shown in overweight subjects [11], may lend clinical support to a cardioprotective effect of PTX3 and further suggests an adverse effect of low PTX3 levels in GDM on early atherosclerotic progression. Tombetti et al. [39] found increased PTX3 in patients with Takayasuartiritis and increased vascular inflammation. Recently, Camozzi et al. [40], demonstrated a protective role of PTX3 after vessel injury, limiting intimal thickening. Taken together, this suggests that there is a contradiction between a condition involving vascular inflammation (e.g. overt atherosclerosis, large-vessel vasculitis, and pre-eclampsia) and its association with metabolic variables (lipid profiles, insulin resistance and β-cell function, BMI) and, as mentioned above, in a vascular inflammatory condition PTX3 might be enhance a protective feedback mechanism activated by inflammation and vascular injuries. While some anti-diabetic drugs has been shown to have beneficial effects on markers of vascular inflammation [41] and PTX3 decreased after 24 weeks of administration of sitagliptin [42], the effects of anti-diabetic treatment on PTX3 in GDM would be of interest. It is clear that the regulation of PTX3 in pregnancy is complex, and the different profiles observed in GDM and preeclampsia patients may reflect contributions from different sources depending on the presence and extent of acute or chronic inflammatory conditions.

### PTX3 in early pregnancy as a predictor of future CV risk

At 5-year follow-up, women with GDM showed an unfavorable lipid profile, with low levels of HDL-C and apoA and high levels of TG, as also demonstrated by others [43]. We have previously reported enhanced TG/HDL-C ratio as a marker of cardiometabolic risk in women with a history of GDM [29]. In the present study we showed that at 5-year follow-up, PTX3 levels correlated well with apolipoprotein levels. Low serum PTX3 levels were particularly prevalent in patients with an enhanced apoB/apoA ratio, representing a pro-atherogenic lipid profile. Importantly, when investigating PTX3 in pregnancy as a predictor of the apoB/apoA ratio at 5-year follow-up, we found that PTX3 was associated with an increase in this ratio as early as 14–16 weeks of gestation, and this association remained after adjusting for BMI and systolic BP. This suggests that the effects of low PTX3 on CVD risk do not merely reflect a different body composition. One limitation of our study is that we did not have standard lipid profiles at the different timepoints in pregnancy. Also, as the women in our study were young with no CVD events, a longer follow-up time would reveal if low PTX3 in pregnancy was associated with actual CV events.

### Potential mechanisms

**Fig. 3:**
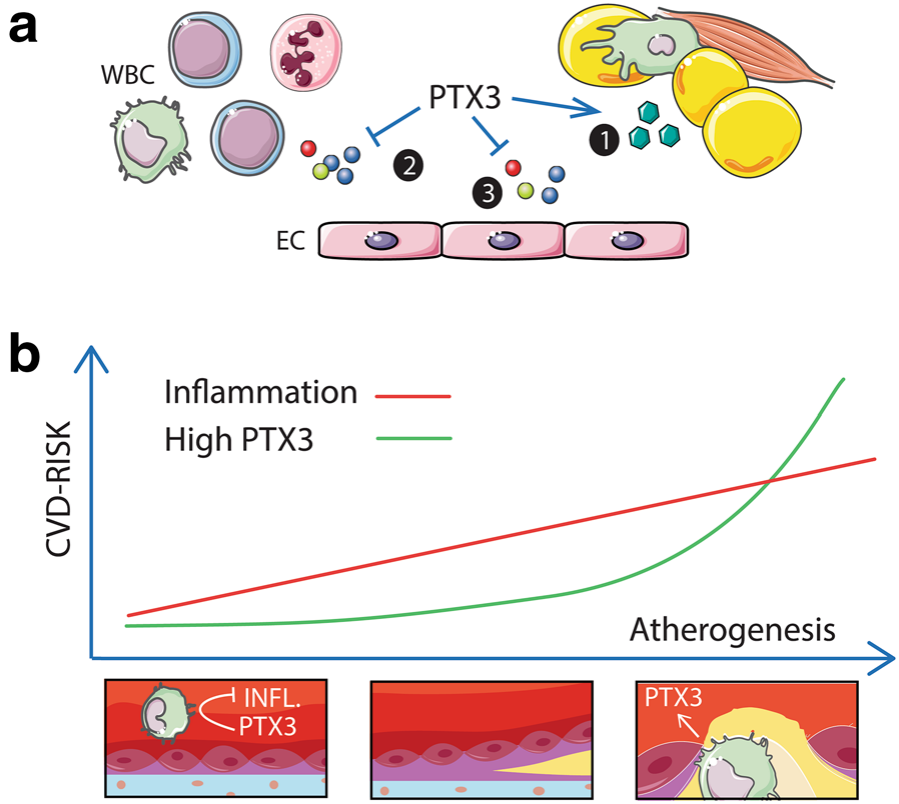
Model integrating the cardio-metabolic effects of low PTX3 in GDM. **a** In the presence of obesity and low grade inflammation, *1* low PTX3 may enhance insulin resistance through diminished effects on glucose transport proteins. *2* Decreased PTX3 may enhance complement mediated inflammation and promote recruitment of leukocytes to inflamed endothelium. *3* Low HDL-C may suppress PTX3 expression and release from endothelial cells (EC). **b** Although these effects may not result in manifest CVD disease they may associate with an enhanced risk profile post partum and possible clinical disease in the long run. In the presence of advanced atherogenesis and incident CVD, a compensatory increase in PTX3 will be associated with adverse outcome

Although the precise determinants of PTX3 levels in pregnancy remain unknown, a range of metabolic risk factors were shown to be independent predictors of PTX3 at 5-year follow-up. As such, the low levels of PTX3 early in pregnancy in women with GDM are likely the result of a composite of metabolic derangements. HDL-C induces PTX3 expression in endothelial cells in vitro [44], and HDL-C levels reportedly decrease in GDM as early as the second trimester [45]. The lack of association with CRP supports that a potential beneficial effects of PTX3 may be independent of its anti-inflammatory effects. It is possible that low HDL-C levels could contribute to low PTX3 levels which again may contribute to an unfavorable lipid profile. Amongst several functions, HDL-C may promote cholesterol efflux from peripheral tissues and transport it to the liver for excretion, thus protecting against CVD. Although our clinical data do not permit any mechanistic insight into the role of PTX3 in pregnancy and GDM, we nonetheless speculate on its potential role by integrating the cardio-metabolic effects derived from experimental studies and clinical observations as described above in Fig. 3.

In conclusion, our results show that plasma PTX3 concentrations in women with GDM are significantly lower than those in non-GDM women, both in early pregnancy and at 5-year follow-up. Further, circulating PTX3 levels in early pregnancy can predict CVD risk using the apoB/apoA ratio as a proxy at 5 years postpartum. Larger prospective studies are needed to investigate the mechanism by which PTX3 levels are altered in women with GDM and to evaluate its prognostic potential for future CVD risk.

## Additional file

10.1186/s12933-016-0345-1 **Table S1.** Plasma levels of PTX-3 and CRP during and after pregnancy in normal and GDM pregnancy according to IADSPSG criteria. **Table S2.** Univariate associations for increased CV risk as reflected by the apoB/apoA, LDL and TG/HDL-C ratios by PTX3, CRP and other CV risk factors at 5 years follow-up. **Table S3.** Multivariable adjusted models for increased CV risk as reflected by the apoB/apoA, LDL and TG/HDL-C ratios by PTX3 during pregnancy. The adjusted analysis included BMI and systolic BP acquired at the same time as the PTX3 measurement. **Figure S1.**  ROC curves of  ApoB/apoA and LDL/HDL-C during various time-points in pregnancy and CV risk at 5 years after pregnancy as reflected by the apoB/apoA ratio at 5 years follow-up.

## Abbreviations

Apo: apolipoprotein
BMI: body mass index
CRP: C reactive protein
CVD: cardiovascular disease
GDM: gestational diabetes mellitus
HDL: high density lipoprotein
HOMA-IR: homeostasis model assessment-insulin resistance
LDL: low density lipoprotein
OGTT: oral glucose tolerance test
PTX3: pentraxin 3
PWV: pulse wave analysis
TG: triglycerides
VAT: visceral adipose tissue
