# Supplementary material for: Low circulating pentraxin 3 levels in pregnancy is associated with gestational diabetes and increased apoB/apoA ratio: a 5-year follow-up study
Source: Cardiovasc Diabetol. 2016 Feb 3;15:23. doi: 10.1186/s12933-016-0345-1 (PMC4739426; doi:10.1186/s12933-016-0345-1)

Supplementay file Lekva et al.

**Table S1.** Plasma levels of PTX-3 and CRP during and after pregnancy in normal and GDM pregnancy according to IADSPSG criteria

|  | PTX-3 | |  | CRP | |
| --- | --- | --- | --- | --- | --- |
| Week | GDM | Non-GDM |  | GDM | Non-GDM |
| 14-16 | 2.70  (1.84, 3.93) | 3.29  (2.38, 4.59) |  | 2.01*  (2.19, 3.16) | 1.57  (0.88, 2.39) |
| 22-24 | 3.03  (2.26, 4.27) | 3.49  (2.54, 4.79) |  | 2.37**  (1.54, 3.86) | 1.55  (0.91,2.47) |
| 30-32 | 3.76  (2.89, 5.35) | 4.26  (3.01, 5.71) |  | 2.05**  (1.09, 2.68) | 1.37  (0.82, 2.16) |
| 36-38 | 6.14  (4.88, 8.05) | 6.33  (4.58, 8.89) |  | 1.73  (1.03, 2.52) | 1.33  (0.80, 2.32) |
| 5 yr  follow-up | 1.51 *  (0.97, 3.41) | 2.29  (1.38, 3.91) |  | 0.45  (0.27,1.16) | 0.38  (0.18, 0.72) |

Median (25^th^, 75^th^) *p<0.05, **p<0.01 vs. Non-GDM

**Table S2.** Univariate associations for increased CV risk as reflected by the apoB/apoA, LDL and TG/HDL-C ratios by PTX3, CRP and other CV risk factors at 5 years follow-up.

|  | apoB/apoA>0.59 | apoB/apoA>0.79 | LDL/HDL>3.0 | TG/HDL>1.09  OR (95% CI) p-value |
| --- | --- | --- | --- | --- |
|  | OR (95% CI) p-value | OR (95% CI) p-value | OR (95% CI) p-value | OR (95% CI) p-value |
| PTX3 (log per SD) | 1.93 (1.37-2.71) <0.001 | 5.36 (2.28-12.59) <0.001 | 4.40 (2.19-8.85) <0.001 | 2.27 (1.37-3.76) 0.001 |
| CRP (log per SD) | 1.28 (0.93-1.75) 0.126 | 1.38 (0.65-2.94) 0.402 | 1.37 (0.75-2.52) 0.308 | 1.38 (0.84-2.27) 0.204 |
| Age (years) | 1.01 (0.93-1.09) 0.800 | 0.90 (0.74-1.10) 0.290 | 0.92 (0.78-1.07) 0.278 | 0.94 (0.83-1.07) 0.342 |
| Family history heart disease | 1.32 (0.69-2.51) 0.402 | 0.92 (0.20-4.18) 0.913 | 1.22 (0.35-4.26) 0.758 | 1.29 (0.46-3.58) 0.632 |
| Parity | 0.73 (0.30-1.80) 0.780 | 0.16 (0.03-0.75) 0.020 | 0.21 (0.06-0.74) 0.016 | 0.58 (0.16-2.14) 0.414 |
| Smoking | 1.52 (1.04-2.22) 0.031 | 1.95 (0.81-4.69) 0.137 | 1.52 (0.74-3.11) 0.257 | 1.05 (0.56-1.99) 0.871 |
| BMI (log per SD) | 1.67 (1.23-2.25) 0.001 | 1.74 (0.91-3.30) 0.093 | 1.95 (1.16-3.28) 0.011 | 3.27 (2.01-5.30) <0.001 |
| SBP (log per SD) | 1.26 (0.92-1.71) 0.150 | 2.88 (1.27-6.57) 0.012 | 2.92 (1.49-5.75) 0.002 | 2.53 (1.47-4.36) 0.001 |

**Table S3.** Multivariable adjusted models for increased CV risk as reflected by the apoB/apoA, LDL and TG/HDL-C ratios by PTX3 during pregnancy. The adjusted analysis included BMI and systolic BP acquired at the same time as the PTX3 measurement.

|  |  | 14-16 weeks | 22-24 weeks | 30-32 weeks | 36-38 weeks |
| --- | --- | --- | --- | --- | --- |
|  |  | OR (95% CI) p-value | OR (95% CI) p-value | OR (95% CI) p-value | OR (95% CI) p-value |
| apoB/apoA | UNI | 1.50 [1.04-2.16] 0.026 | 1.61 [1.13-2.20] 0.009 | 1.51 [1.07-2.14] 0.019 | 1.41 [1.02-1.96] 0.038 |
| >0.59 | ADJ | 1.53 [1.07-2.20] 0.020 | 1.46 [1.01-2.11] 0.043 | 1.39 [0.97-1.99] 0.069 | 1.30 [0.93-1.82] 0.125 |
|  |  |  |  |  |  |
| apoB/apoA | UNI | 3.26 [1.52-7.00] 0.002 | 3.12 [1.31-7.42] 0.010 | 2.56 [1.17-5.59] 0.018 | 1.76 [0.84-3.66] 0.132 |
| >0.79 | ADJ | 2.79 [1.13-6.85] 0.026 | 2.79 [1.13-6.85] 0.026 | 2.27 [1.00-5.13] 0.050 | 1.60 [0.71-3.6]0 0.253 |
|  |  |  |  |  |  |
| LDL/HDL | UNI | 2.60 [1.38-4.91] 0.003 | 2.54 [1.26-5.11] 0.009 | 2.04 [1.07-3.90] 0.031 | 1.74 [0.96-3.17] 0.069 |
| >0.79 | ADJ | 2.34 [1.23-4.47] 0.010 | 2.27 [1.08-4.75] 0.030 | 1.77 [0.91-3.47] 0.095 | 1.58 [0.84-3.00] 0.158 |
|  |  |  |  |  |  |
| TG/HDL | UNI | 1.69 [0.96-2.96] 0.068 | 1.61 [0.89-2.93] 0.119 | 1.78 [1.00-3.20] 0.051 | 1.32 [0.77-2.27] 0.311 |
| >1.09 | ADJ | 1.43 [0.80-2.55] 0.228 | 1.30 [0.68-2.48] 0.432 | 1.83 [0.93-3.61] 0.081 | 1.05 [0.57-1.93] 0.876 |

**Figure S1.** ROC curves of ApoB/apoA and LDL/HDL-C during various time-points in pregancy and CV risk at 5 years after

pregnancy as reflected by the apoB/apoA ratio at 5 years follow-up.


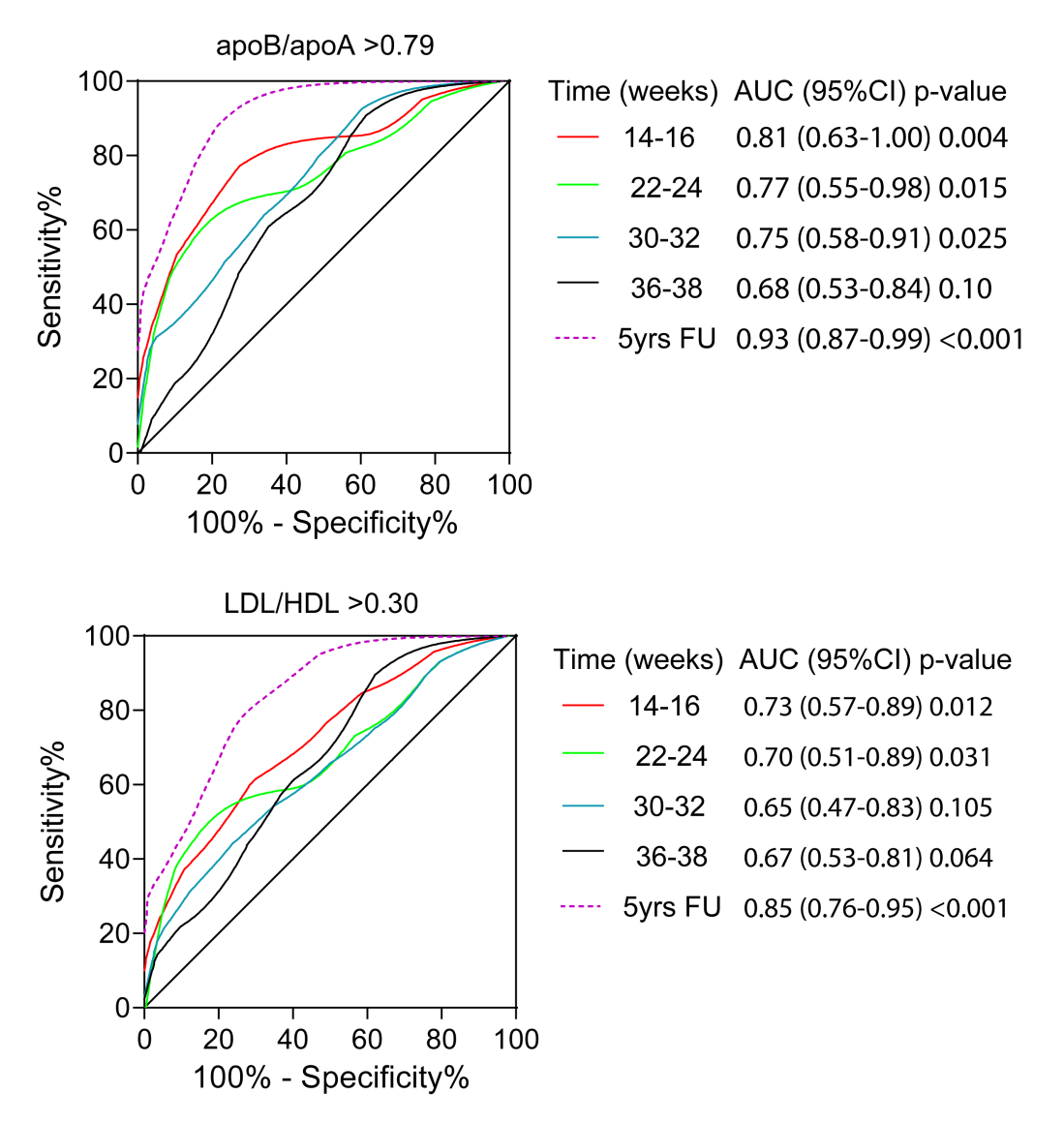

Supplement: Supplementary file 1 — 10.1186/s12933-016-0345-1 Table S1. Plasma levels of PTX-3 and CRP during and after pregnancy in normal and GDM pregnancy according to IADSPSG criteria. Table S2. Univariate associations for increased CV risk as reflected by the apoB/apoA, LDL and TG/HDL-C ratios by PTX3, CRP and other CV risk factors at 5 years follow-up. Table S3. Multivariable adjusted models for increased CV risk as reflected by the apoB/apoA, LDL and TG/HDL-C ratios by PTX3 during pregnancy. The adjusted analysis included BMI and systolic BP acquired at the same time as the PTX3 measurement. Figure S1. ROC curves of ApoB/apoA and LDL/HDL-C during various time-points in pregnancy and CV risk at 5 years after pregnancy as reflected by the apoB/apoA ratio at 5 years follow-up. [file 12933_2016_345_MOESM1_ESM.docx]
